# Stimulating injury-preventive behaviour in sports: the systematic development of two interventions

**DOI:** 10.1186/s13102-019-0134-8

**Published:** 2019-10-21

**Authors:** E. Kemler, H. Valkenberg, V. Gouttebarge

**Affiliations:** 1 Dutch Consumer Safety Institute, Overschiestraat 65, 1062 Amsterdam, XD the Netherlands; 2 Department of Orthopaedic Surgery, Amsterdam Movement Sciences, Amsterdam UMC, University of Amsterdam, Meibergdreef 9, Amsterdam, The Netherlands; 3 Amsterdam Collaboration for Health & Safety in Sports (ACHSS), AMC/VUmc IOC Research Center of Excellence, Amsterdam, The Netherlands; 4 Division of Exercise Science and Sports Medicine (ESSM), University of Cape Town, Cape Town, South Africa

**Keywords:** Skiing, Snowboarding, Running, Intervention, Injury-preventive behaviour, Intervention mapping, Knowledge transfer scheme, Expert-driven approach

## Abstract

**Background:**

In addition to the beneficial health effects of being active, sports are also associated with a risk of sustaining injuries. To avoid the occurrence of sports injuries, preventive measures can be applied. The aim of the current article is to provide insight into the systematic developmental process of two evidence-based interventions designed to stimulate injury-preventive behaviour in runners and skiers, in which Intervention Mapping (IM) and Knowledge Transfer Scheme (KTS) are used as developmental protocols. However, the ultimate steps in the process are adjusted to meet requirements of the intervention and the target group.

**Methods:**

Using a three-step process, we developed two interventions to stimulate injury-preventive behaviour in runners and skiers. Sports participants, sports experts and behaviour experts contributed throughout steps two and three of the developmental process.

**Results:**

In step one we started with a problem statement in which we used information about the number and the burden of running-related and skiing-related injuries in the Netherlands. In step two, in-depth research was performed using four research strategies. During this step we tried to answer the following question: *Which preventive measures or actions should be executed to prevent what injuries by whom, and how should we do that?* A desk research/systematic review of the literature, expert meetings, target user surveys, and target user focus group meetings were conducted. In step three of product development, both interventions were developed. During the developmental process, co-creation sessions with target users were held. Before finalizing the interventions, pre-tests of the interventions were performed with target users.

**Conclusions:**

Through a three-step approach, we developed two interventions to stimulate injury-preventive behaviour in runners and skiers. To develop an intervention that fits the needs of the target population, and will be used by them, it is necessary to involve this population as soon and as much as possible. Several steps in the IM and KTS protocols have thus been adjusted in order to establish an optimal fit between intervention and target group.

## Background

Running and winter sports like skiing and snowboarding (hereafter referred to as skiing) are popular forms of exercise among the Dutch population [1, 2]. While being active through running and skiing has undeniable beneficial health effects, both sports are also associated with a high risk of musculoskeletal injuries [3–9]. Additionally, skiers are at a high risk for traumatic brain injury [7–9]. In both sports, the level of expertise is one of the associated risk factors for injuries [4, 10, 11].

To safeguard (novice) sports participants against injuries, and consequently from withdrawal from their activities, the use of effective injury-preventive measures is necessary. The use of effective measures often requires a behavioural change in sports participants. For some sports, the use of effective injury-preventive measures, such as helmets in bicycle races or shin guards in football, have been made compulsory. The enforcement of these compulsory measures and the risk for (external) penalties are motivating factors to stimulate injury-preventive behaviour. However, in the case of running, the implementation of compulsive injury-preventive measures is difficult, and none exist for adult skiers. Therefore, significant efforts have to be made to accomplish a behavioural change in order to increase the use of effective measures and subsequently reduce the number of running-related and skiing-related injuries.

With running, some interventions have been recently implemented, leading to promising results in stimulating injury-preventive behaviour and preventing running-related injuries (RRIs) [12, 13]. Hespanhol et al. [12] observed no effects on determinants and actual preventive behaviour, but RRIs were prevented. An intervention developed by Adriaensens et al. (Dutch Consumer Safety Institute) was effective in stimulating injury-preventive behaviour among runners [13], but was very time-consuming.

To prevent injuries or reduce injury risk in skiing, several educational interventions have been used in the past [14, 15]. According to a systematic review by Hume et al. [10], the effectiveness of educational interventions is unclear due to the diverse nature of the education campaigns and target populations, but could possibly be beneficial.

Although several interventions have therefore been recently developed for runners and skiers, it is, to our knowledge, unknown how these interventions were developed. Insight into the developmental process of an intervention can provide knowledge and practical guidance for others in their developmental process [16]. In terms of the development of interventions concerning injury prevention in sports, several development protocols can be used, including Intervention Mapping (IM) [17, 18] and the Knowledge Transfer Scheme (KTS) [19]. The IM protocol describes the iterative path from problem identification to problem solving or mitigation. Each of the six steps of IM comprises several tasks, each of which integrates theory and evidence [17, 18]. The KTS integrates existing implementation research frameworks into a tool which has been developed specifically to bridge the gap between knowledge derived from research on the one hand and evidence-based usable information and tools for practice on the other [19]. Both IM and KTS rely on consecutive steps and are closely related to each other. There are, however, key differences. For example, the KTS uses target users throughout the entire development process, whereas IM only uses them during the needs assessment stage [17–19]. IM focuses more on investigating behavioural change determinants and developing new strategies [17, 18], whereas the aim of KTS is to translate evidence into practice [19].

The IM and KTS protocols act as a guide to developing interventions, but sometimes adjustments in the developmental process have to be made, for example due to practical issues. The aim of the current article is to provide insight into the systematic developmental process of two evidence-based interventions to stimulate injury-preventive behaviour in runners and skiers, in which IM and KTS are used as a developmental protocol (i.e. an overview of aspects to be covered in the development of an intervention). However, the final steps in the process are adjusted to meet the requirements of the intervention and the target group.

## Development of the interventions

**Fig. 1:**
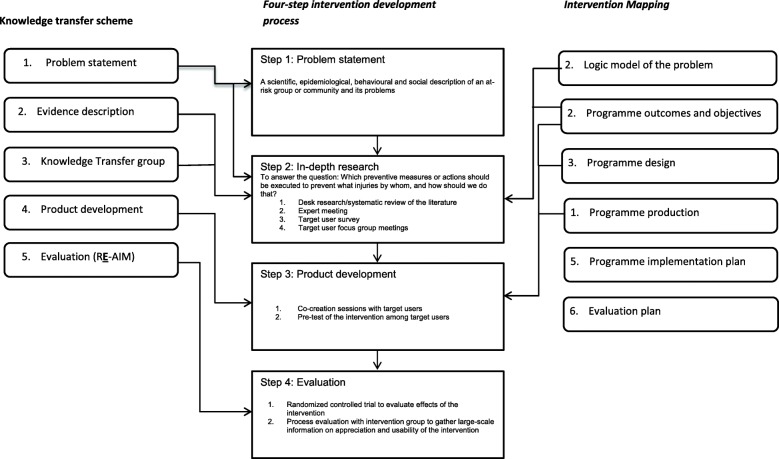
Flow chart of the four-step development process

As stated above, both IM (six steps) and KTS (five steps) rely on consecutive steps and are closely related to each other. To develop two interventions to stimulate injury-preventive behaviour in runners and skiers, we combined steps from IM and KTS in a systematic four-step approach (see Fig. 1). The consecutive steps were based on two basic dimensions: 1) the close involvement of the target group (sport participants) and experts in the development of the intervention; and 2) the range of available methods for collecting relevant input and content for the development of the intervention, with separate research methods collecting information for different steps in the process. During desk research, expert meetings, survey and group interviews with the target group, information was simultaneously collected for problem statement, risk factors, intervention development, and the like.

Steps one to three are related to the problem exploration and the development of an intervention. Sports participants, sports experts, researchers and behaviour experts contributed throughout the developmental process. In step four, the effects of the intervention are evaluated in a randomized controlled trial.

### Step 1: problem statement

The starting point of the development of our interventions was a potentially effective, but time-consuming – and therefore unattractive and complex – intervention for injury prevention in running [13] and a problem statement for both sports identical to the first component of the problem statement in KTS. We used information on the number and severity of running-related and skiing-related injuries in the Netherlands, and calculations of financial costs of injuries, in an epidemiological analysis of the problem. Both the number and risk for RRIs in the Netherlands was high, while skiing-related injuries were often severe [20].

### Step 2: in-depth research

As the number and the burden of injuries do not provide sufficient information to guide or start a development process, we undertook several research approaches to collect the required additional information. In this step, we strived to answer the following question: Which preventive measures or actions should be executed to prevent which running-related and skiing-related injuries by whom, and how should we do that?

We used four preferred research approaches among different target groups to answer this question:

i) *review of scientific literature* to explore the most recent developments in injury prevention in running and skiing, and the mechanisms behind the effectiveness of these injury-preventive measures. Furthermore, information on existing interventions was collected. In March 2016, articles published in PubMed after the year 2000 were searched, using the following terms: injury*, prevent*, running, skiing, snowboard, effect*, intervent*, measure. Reviews, RCTs, and prospective studies were included.
ii) *expert meetings* (one with running experts and one with skiing experts) were conducted in April 2016, to target high-risk groups and to prioritize injuries and injury preventive measures. During these 2-h sessions, the scientific literature on the incidence, prevalence, aetiology, risk factors and preventive measures of injuries was discussed, alongside strategies for injury prevention. Sport-specific experts were recruited by the Royal Dutch Running Association (KNAU) and the Dutch Skiing Association (NSkiV); they were able to claim financial compensation for their invested time. The running expert group consisted of a sports physician, a sports physiotherapist, two running coaches and a researcher in sports injuries. The skiing expert group consisted of three sports physiotherapists/skiers, two employees of the NSkiV and a researcher in sports injuries. Discussion leaders facilitated the expert meetings. Written reports of the meetings (topics, questions, answers and conclusions) were used as input information during the actual development of the intervention. No specific qualitative data analysis programme was used.
iii) *quantitative study among target populations (717 runners, 283 skiers)*: an anonymous one-time online survey was distributed among both target populations (running and skiing) in May 2016 and administered for 2 weeks. The respondents were recruited through social media accounts (Facebook, Twitter, LinkedIn) from the KNAU, the NSkiV, Runner’s World magazine and the Dutch Consumer Safety Institute. Respondents who completed the questionnaire were entered into a draw in which they could win a 1-year subscription to Runner’s World magazine (five subscriptions in total). The online survey was set up to explore whether the (qualitative) information collected through the expert meetings was largely supported by the actual target groups for prevention. Questions were formulated in relation to, among others: (i) RRIs and skiing injuries; (ii) current and future injury-prevention behaviour in running and skiing; (iii) determinants of injury-preventive behaviour; (iv) needs and support for the intervention; (v) characteristics (content, form) of the intervention; and (vi) effective strategies to implement the intervention. Descriptive statistics, in SPSS version 23, were used to analyse the data from the online survey.
iv) *focus group meetings with target populations* were conducted in order to establish the best ways to approach runners and skiers for preventive actions. Two 2-h meetings were organized with runners, one meeting with four runners and one meeting with five. Focus group meetings (2 h per meeting) for skiing also consisted of four and five participants. The participants were recruited through the online questionnaire of the quantitative study. At the end of this questionnaire, respondents were asked whether they would like to participate in focus group meetings. Participants were randomly selected from a list of respondents willing to participate in further research. Focus group meetings were held in June/July 2016. All participants received a gift voucher to the value of €20. As with the expert meetings, the focus group sessions were facilitated by two discussion leaders. Written reports of the meetings (topics, questions, answers and conclusions) were used as input information during the actual development of the intervention. No specific qualitative data analysis programme was used.

As a result of this step two of in-depth research, consisting of different methods of data collection, a development plan for both interventions was established, containing information on the requirements of the interventions and an overview of the behaviour determinants to be tackled, and theories and strategies to be applied.

Main preferences and requirements for the interventions were:

- A focus on a specific type or specific types of running or skiing injury was not desirable.
- The two main general risk factors in running injuries to be tackled are: 1) *the amount of stress or force* a person can impose on the body (based on fitness, body weight, sports history, etc.) as evidence shows that training errors or training overload can lead to half of all injuries [21, 22]; and 2) goal-setting behaviour of the athlete (e.g. running 5 km within 6 weeks, running a marathon; [Romeijn, Kemler, Huisstede; submitted]). In skiing, the general risk of sustaining a skiing injury was mainly a result of: 1) a skier’s physical fitness (e.g. muscular strength, endurance, dealing with fatigue [15]); and 2) technical skiing ability [10].
- A few easily accessible and simple preventive measures should be proposed for injury prevention, in order to minimize the threshold for performing the target behaviour.
- The intervention objective were to positively change injury-preventive behaviour in the following main target groups: 1) novice runners [4]; 2) novice skiers [10, 11]; and 3) recreational (holiday) skiers [23].
- The main preventive measures proposed for runners were: using a *training schedule* that is appropriate for the physical condition of the athlete and represents a realistic running goal; doing *strength exercises* [24] to improve the amount of force that can be exerted on the body while running; and an *active warm-up* prior to running [25].
- Important preventive measures proposed for skiers were: performing s*trength exercises* in the period prior to the skiing vacation to improve strength, physical fitness and endurance [26]; improving *technical skills*; using a *skiing helmet* [27–31]; increasing knowledge of ski slope regulations (the Fédération Internationale de Ski (FIS) regulations) [32].
- Finally, the interventions should be accessible by smartphone, tablet and PC.

### Step 3: product development

**Fig. 2:**
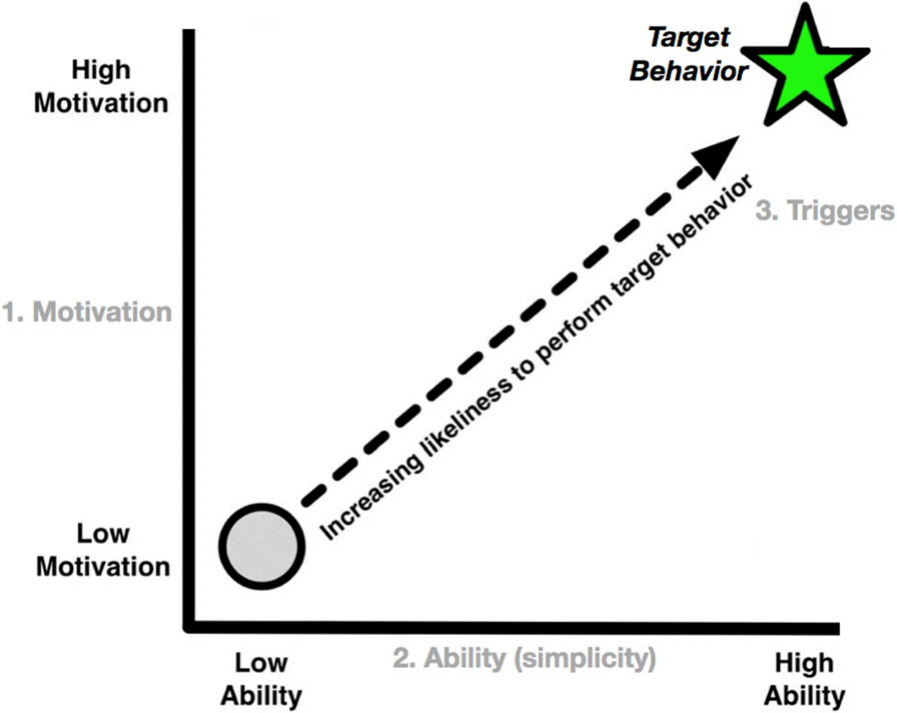
The Fogg Behaviour Model has three factors: motivation, ability, and triggers. Reprinted from [33]

The interventions were developed in 11 months. The review of the literature started in April 2016. The final adjustments to the interventions, after the pre-test, were made in February 2017. The development of the interventions was supervised by a company specialised in behaviour change, experts in simplifying the choices people have to make, and offering easy steps for behavioural change by means of a creative, intuitive and visually attractive concept. Their vision and the development of the intervention was based on the Fogg Behaviour Model (FBM) to achieve behaviour change. The FBM model is mainly used in the field of persuasive technology. FBM views behaviour as a product of three factors: *motivation*, *ability* and *triggers* (Fig. 2) [33]. All three factors must ideally be present simultaneously to achieve the required behaviour change. In other words, high motivation, high ability, and triggers will optimize behaviour change.

Based on this model, and on running and skiing experts, the content and form of the intervention were established. The main strategies were to:

- increase Motivation through developing a visually attractive web-based tool that stresses the positive effects of injury prevention on experiencing running and skiing activities, through a focus on the pleasure of performing sports without fearing injuries;
- increase Ability through the simplicity of the web-based tool (a few, easily accessible and easy-to-perform preventive measures), and by tailoring the intervention towards specific subgroups and adjusting the preventive measures to the characteristics and ability of the subgroup.

Additionally, analogous to FBM, triggers have to be included in the intervention to persuade the athletes to actually perform the intended behaviour. Examples of triggers are videos, links to relevant websites, and graphics to stimulate the target behaviour.

The preferred methods and approaches for developing the interventions for runners and skiers were directly derived and translated from theoretical concepts in intervention development (Knowledge, Awareness and Self-efficacy). The results of this theoretical translation are presented in Table 1. The intervention for runners (Runfitcheck) is used as an example.

**Table 1:** Theoretical justification of our approach: description of factors, aims, methods and approaches used in Runfitcheck

Factor	Aim	Method	Description method	Approach in Runfitcheck
Knowledge	To know which injury-preventive behaviours are available.	Knowledge transfer (Schaalma et al., 2001) [34]	Providing of and/or transfer of knowledge on the desired behaviour.	In the advice provided in the intervention, information on injury-preventive behaviour is transferred to runners.
		Advance organizers (Kools et al., 2006) [35]	Presenting an overview of the material that enables a learner to activate relevant schemas so that new material can be associated.	The advice on injury-preventive behaviour is presented in an accessible way with clear headings. Furthermore, runners can receive a personal schedule for running sessions and strength exercises each week (by email). The schedules are accompanied by instructions on how to carry out the strength exercises or running session.
		Chunking (Smith, 2008) [36]	Using stimulus patters that may be made up of parts but that one perceives as a whole.	The name of the intervention for runners is Runfitcheck, which refers to the necessity for the runners to prepare themselves for a running session and to verify their physical fitness prior to the running session. The name of the intervention reminds the runners that it is important to pay attention to their physical fitness when they go for a run.
Awareness	To be aware that the execution of injury-preventive behaviour is important to prevent RRIs.	Consciousness raising (Prochaska et al., 2008) [37]	Providing information, feedback or confrontation about the causes, consequences and alternatives for a problem or a problem behaviour.	The intervention informs the runners about their risk for RRIs. For example, it is stated that novice runners do have a higher risk than more experienced runners Also, runners gain insight into the amount of stress or force they can put on their body, and goal-setting behaviour.
		Knowledge transfer (Schaalma et al., 2001) [34]	Providing of and/or transfer of knowledge on the desired behaviour.	The transfer of information on injury-preventive behaviour increases the runners’ awareness of that injury-preventive behaviour.
Self-Efficacy	To feel able to execute injury-preventive behaviour.	Guided practice (McAlister et al., 2008) [38]	Prompting individuals to rehearse and repeat the behaviour various times, discuss the experience, and provide feedback.	In the intervention, runners are stimulated to execute injury-preventive behaviour by clear instruction in the advices, and instruction videos guided by voice over These instruction videos are also available in the personal schedules for running sessions and strength exercises which runners can receive each week (by email).
		Modelling (Bandura,1997 [39]; McAlister et al., 2008 [37]; Rogers, 2003) [40]	Providing an appropriate model to reinforce the desired action.	In the intervention, several ordinary athletes execute the desired injury-preventive behaviours. These athletes give a good example, e.g. they perform warm-up routines or strength exercises.
		Tailoring (Lustria et al., 2009) [41]	Matching the intervention or components to previously measured characteristics of the participants.	In the intervention, the advice given is based on a runner’s physical fitness (th*e* amount of stress or force the runner can impose on his body) and goal-setting behaviour, which are assessed at the start of the intervention.

In addition to cooperating in the in-depth research steps of the process, the target groups of runners and skiers (i.e. the actual athletes) took part in the actual development of the interventions, alongside the research team, experts, and the company specialized in interventions to change behaviour. *Co-creation sessions with the target population* were held to discuss the first concepts of the interventions. Runners and skiers were invited to comment on content, visuals, and the design of the intervention. A 2-h meeting was organized with three runners, and a 2-h session was organized with three skiers. The participants were recruited from the focus group sessions. The main focus in these sessions was to link intervention and target group in order to maximize the possibility of athletes actually increasing injury-prevention behaviour.

The comments of the runners and skiers were used to fine-tune and optimize the interventions.

Then, *a pre-test of the intervention* was held after the interventions were optimized*.* To recruit the athletes, an email was sent to contact persons of the Dutch Consumer Safety Institute who were involved in running or skiing, and the participants of the quantitative studies. Those who were interested were given access to the interventions and filled in a questionnaire on how they experienced the intervention. Seventeen runners and 17 skiers provided their feedback. The interventions (named Runfitcheck and Wintersportklaar) were finalized using their feedback.

Since part of the starting point of the development of our interventions was a tailor made, potentially effective – but lengthy and complex – intervention for injury prevention in running [13], one of the goals was to develop an alternative intervention in which differentiation of target groups was a main objective. This approach was supported by the experts during the expert meetings, with a preference for basic, easy access to the intervention. As a result, a short questionnaire was proposed by the experts, as a simple means of distinguishing target groups for tailoring prevention, and this was developed by the company specialised in developing interventions to change behaviour together with researchers from the project group. This short questionnaire was evaluated several times by athletes during focus group sessions, co-creation sessions and pre-testing, and adapted to athletes’ preferred way of determining their own (subjective) fitness/experience/vulnerability/motivation. Runners fill in four questions on their “fitness” and two on their “goal-setting behaviour”. Skiers answer three questions on their physical fitness and three questions on their technical ability. Based on the answers, athletes are allocated to one of four basic quadrants, for example “low fitness/high ability” in skiing.

After completing the questionnaire, and subsequently being informed of the specific quadrant an athlete has been allocated to, the athletes receive a range of advice on injury prevention.

The experts in the project team provided the actual preventive measures for the intervention. Measures were differentiated for different quadrants, i.e. tailored towards different subgroups of the target group. Runners and skiers in different quadrants received different running schemes, schedules for strength exercises, technique tips, and the like. Participants were additionally provided with the opportunity to subscribe to even more personalized training advice via weekly emails, based on the number of weeks preparation before a running event or skiing vacation.

## Discussion

Using a three-step process – an abbreviation or moderation of the various steps from Intervention Mapping and Knowledge Transfer Scheme protocols – we developed two interventions to stimulate injury-preventive behaviour in runners and skiers. Sports participants, sports experts and behaviour change experts contributed throughout steps two and three of the developmental process.

Our methodology to develop the interventions was guided by IM and KTS. As mentioned by Pas et al. (2018) [42], (strictly) following a protocol might pose some problems as, in this case, sports-specific practices might differ for different types of sports. It is important to use a protocol to guide your process, and to keep track of the important steps to be taken. It is equally important, however, to keep your specific population and situation in mind too. We started our developmental process with a problem statement. The next step was an in-depth research. Our step of in-depth research is extensive, and closely related to steps one to three from the KTS and steps one and two of IM. The expert meeting is comparable to the Knowledge Transfer Group in KTS. The experts were all runners or skiers and coaches or physiotherapists, and we discussed the problem statement, scientific evidence and the development of the required intervention with them. Furthermore, we asked them to verify the scientific evidence against the current situation in their type of sport. For example, according to the evidence for skiing, the use of a winter sport helmet should be stimulated. However, today it is normal for Dutch inhabitants to wear winter sports helmets while skiing. Although it is still important to mention the use of a winter sport helmet, stimulating this injury-preventive measure should not be the main focus of our injury-preventive intervention.

Sports participants were given an important role in this developmental process. From our developmental experiences in the past with regard to injury-preventive interventions, we know that if participants cannot relate to the intervention due to the content, its form or even tone of voice, they will not execute the intervention. Therefore, we did not only consult them in step one of IM/KTS (problem statement/needs assessment), but we also used the online questionnaire to explore whether, amongst other things, the (qualitative) information collected through the expert meetings was largely supported by the sports participants. Furthermore, we collected information on their current and future injury-prevention behaviour in running and in skiing, their needs and support for an intervention, and the characteristics and deliver strategies of such an intervention.

With the use of KTS and IM, we designed our own developmental process. Donaldson et al. (2016) published a systematic but pragmatic and iterative intervention development process as well [16]. Their six-step intervention development process involves: (1) compiling research evidence, clinical experience, and knowledge of the implementation context; (2) consulting with experts; (3) engaging with end users; (4) testing the intervention; (5) using theory; and (6) obtaining feedback from early implementers. All these steps are integrated into our three-step process too. Both the processes described underline the idea that research evidence alone is not enough to develop implementable interventions or to change behaviour, and evidence-based practice integrates the best available scientific evidence with practitioner and end user values. The development of an intervention in co-creation with experts and end users clarifies the need for tailor-made interventions. A “one-size-fits-all” intervention will not fulfil the needs of different groups of end users. Hence it is necessary to focus on specific target groups to enhance the usability of an intervention.

In step four of our model, the intervention is evaluated. Both IM and KTS recommend an evaluation of the developed intervention. In IM, after the programme production, a programme implementation plan has to be developed (step five of IM). Finally, in step six an evaluation plan has to be developed. Following the implementation of the intervention, the evaluation will take place. In KTS, step five consists of an evaluation according to the RE-AIM (Reach, Effectiveness, adoption, Implementation, Maintenance) framework [43]. After the development of the interventions, we choose to evaluate the effects of the interventions in a randomized controlled trial. If the interventions are effective in stimulating injury-preventive behaviour, an implementation and evaluation plan for each intervention will be developed (Steps five and six of IM), followed by a large-scale implementation of the interventions (and an evaluation of these implementations).

According to the IM procedures, tailoring the intervention towards specific subgroups of the target population increases the effectiveness of preventive advice to these specific subgroups [17, 18]. In the case of these two interventions, the initial tailoring is restricted to a basic division of the athletes into four quadrants, based on a few questions. Arguably, this can be evaluated as being too simplistic to actually distinguish between target groups. However, because it was a goal of the project to include as many runners and skiers in the group of users, the threshold for participating was kept as low as possible, and an initial questionnaire should not be so comprehensive that athletes drop out before any preventive advice can be offered. In the same way, the preventive advice is not too complex or time-consuming, with only three or four basic options. The rationale behind this choice is that if many athletes use a few simple preventive measures, the effect on total preventive behaviour in a population will possibly be much higher than if only a few athletes perform complex, time-consuming injury-prevention strategies. However, the option to subscribe to personalized training schedules and exercises enhances the intervention, thereby also making it a useful tool for athletes who are willing to invest a little more time and effort in injury prevention. This option provides easily accessible and easy-to-perform actions as well. Arguably, the effort of reducing the threshold for participating in this preventive behaviour through a short questionnaire – and thus maximizing compliance – might result in a less strict division of subgroups than would ideally be the case. It should be added, however, that athletes who use the intervention are provided with the opportunity to change their assigned quadrant if they feel that they have not been allocated to the right quadrant on the basis of the short questionnaire. Ultimately, an effect study should establish whether this basic tailored advice is sufficient to increase preventive behaviour.

During the expert sessions, it was indicated that a focus on a specific type of running or skiing injury was not desirable. This was supported by the review of the literature conducted. While there was no reason to focus attention on specific types of injury, there was evidence for specific risk factors for sustaining a running or skiing injury. The decision not to focus on specific injury types has also been made in the perspective of maximizing compliance: the simplicity of the intervention was a main objective. The most important factors governing injury-free skiing (fitness, ability, regulations) mainly prevent *falls* and *collisions* – injury mechanisms that result in a wide variety of injury types. The most important measures in running (fitness, strength, warm-up) prevent many overuse injuries, irrespective of the specific body type involved. The exception was the recommendation of wearing a helmet while skiing, an evidence-based measure to prevent serious brain damage. Although thoughtful deliberation between the experts took place to balance inclusiveness versus simplicity in this intervention, there may be other relevant injury mechanisms in both sports which are currently underrepresented in this intervention. However, in the experts’ opinion, a focus on fitness and ability (and subsequently on exercises and technique training) would have a bigger impact on a broad range of injuries than a focus on specific types of injuries.

A major challenge in the development of the intervention has been the focus on injury-preventive behaviour as an outcome in the study, instead of the more traditional measurement of injuries. It is well-known that people find it difficult to change health-related behaviour. It is a lengthy process and requires small steps [44]. This intervention was based on the FBM. It is expected that the model’s focus on attractiveness and simplicity to increase motivation and ability, and the addition of triggers to help the athlete to actually perform the desired behaviour, will facilitate behavioural change. A study on the effect of the intervention is needed to establish the magnitude of this change.

It has been stated earlier that running and skiing were selected to assist in developing an injury-prevention tool because of their unorganized nature of participation. This means that there are no intermediate target groups, like coaches or trainers, that help increase the athletes’ compliance to preventive measures. Therefore, a focus on increasing compliance is key, and simplicity and attractiveness of the web-based tool are of utmost importance.

According to Van Mechelen’s ‘sequence of prevention’ model, the development of the online injury-prevention tools for running or skiing should be followed by an evaluation of its effectiveness [45]. Consequently, two prospective controlled trials have been started in the Netherlands in order to establish whether the interventions lead to an increase in injury-preventive behaviour among runners and skiers. In the case of empirical evidence for its effectiveness on behaviour, the implementation of the online interventions will be initiated. In that case, a study that establishes the intervention’s effect on actual running and skiing injuries should be conducted.

## Conclusions

This article describes a “real-life” example of the development of an intervention. It describes required steps in the development, gives insight into the methods used and the lengthy, time-consuming development process. Based on IM and KTS protocols, two interventions to stimulate injury-preventive behaviour among runners, skiers and snowboarders have now been developed. However, we believe that it is necessary to involve end users as soon and as much as possible to develop an intervention that fits the needs of the target population, and that will be used by them. Several steps in the IM and KTS protocols have thus been adjusted in order to establish a maximum fit between intervention and target group, and to maximally increase behavioural compliance to injury-preventive measures. Intervention development might benefit from a more loosely structured step-by-step model, in which preferred research methods are leading in collecting required information, which is then used in the further development of an intervention.

## Supplementary information

**Additional file 1.** Questionnaire target population Running.

**Additional file 2.** Questionnaire target population Skiing.

**Additional file 3.** Roadmap Focus group sessions Running.

**Additional file 4.** Roadmap Focus group sessions Skiing.

## Data Availability

Data sharing is not applicable to this article as no datasets were generated or analyzed during the current study.

## Abbreviations

FBM: Fogg behaviour model
FIS: Fédération Internationale de Ski
IM: Intervention Mapping
KTS: Knowledge transfer scheme
RRI: Running-related injury

## References

[1] VeiligheidNL. Hardloopblessures. Amsterdam, VeiligheidNL, 2014. (Downloaded from the website www.veiligheid.nl at March 3, 2015).

[2] Statistics Netherlands (CBS). https://www.cbs.nl/nl-nl/nieuws/2016/51/wintersport-vooral-populair-onder-hogere-inkomens. Accessed on 27 Feb 2015.

[3] Gend RN van, Siem D, van Middelkoop M, et al. Incidence and determinants of lower extremity running injuries in long distance runners: a systematic review. Br J Sports Med. 2007;41:469–80 discussion 480. 10.1136/bjsm.2006.033548 PMC2465455 17473005

[4] Buist I, Bredeweg SW, Bessem B (2010). Incidence and risk factors of running-related injuries during preparation for a 4-mile recreational running event. Br J Sports Med.

[5] Kluitenberg B, van Middelkoop M, Verhagen E (2016). The impact of injury definition on injury surveillance in novice runners. J Sci Med Sport.

[6] Kluitenberg B, van Middelkoop M, Smits DW, Verhagen E, Hartgens F, Diercks R, van der Worp H (2015). The NLstart2run study: incidence and risk factors of running-related injuries in novice runners. Scand J Med Sci Sports.

[7] DeFroda SF, Gil JA, Owens BD (2016). Epidemiology of lower extremity injuries presenting to the emergency room in the United States: snow skiing vs. snowboarding. Injury..

[8] Patrick E, Cooper JG, Daniels J (2015). Changes in skiing and snowboarding injury epidemiology and attitudes to safety in big sky, Montana, USA: a comparison of 2 cross-sectional studies in 1996 and 2013. Orthop J Sports Med.

[9] Ruedl G, Philippe M, Sommersacher R, Dünnwald T, Kopp M, Burtscher M (2014). Current incidence of accidents on Austrian ski slopes. Sportverletz Sportschaden.

[10] Hume PA, Lorimer AV, Griffiths PC, Carlson I, Lamont M (2015). Recreational snow-sports injury risk factors and countermeasures: a meta-analysis review and Haddon matrix evaluation. Sports Med.

[11] Gaudio RM, Barbieri S, Feltracco P, Spaziani F, Alberti M, Delantone M, Trevisiol P, Righini F, Talarico A, Sanchioni R, Spagna A, Pietrantonio V, Zilio G, Dalla Valle R, Vettore G, Montisci M, Bortoluzzi A, Sacco A, Ramacciato G, Pasetti A, Mognato E, Ferronato C, Costola A, Ori C, Avato FM (2010). Impact of alcohol consumption on winter sports-related injuries. Med Sci Law.

[12] Hespanhol Luiz Carlos, van Mechelen Willem, Verhagen Evert (2017). Effectiveness of online tailored advice to prevent running-related injuries and promote preventive behaviour in Dutch trail runners: a pragmatic randomised controlled trial. British Journal of Sports Medicine.

[13] Adriaensens L, Hesselink A, Fabrie M, Brugmans MJP, Verhagen EALM (2014). Effectiveness of a tailored intervention on determinants and behavior to prevent running related sports injuries: a randomizes controlled trial. Schweizerische Zeitschrift für Sportsmedizin und Sporttraummatologie.

[14] Cusimano M, Luong WP, Faress A, Leroux T, Russell K (2013). Evaluation of a ski and snowboard injury prevention program. Int J Inj Contr Saf Promot.

[15] Jorgensen U, Fredensborg T, Haraszuk JP (1998). Reduction of injuries in downhill skiing by use of an instructional ski-video: a prospective randomised intervention study. Knee Surg Sport Traumatol Arthrosc.

[16] Donaldson A, Lloyd DG, Gabbe BJ, Cook J, Young W, White P, Finch CF (2016). Scientific evidence is just the starting point: a generalizable process for developing sports injury prevention interventions. J Sport Health Sci.

[17] Bartholomew LK, Parcel GS, Kok GJ (1998). Intervention mapping: a process for developing theory- and evidence-based health education programs. Health Educ Behav.

[18] Kok G, Gottlieb NH, Peters GJ, Mullen PD, Parcel GS, Ruiter RA, Fernández ME, Markham C, Bartholomew LK (2016). A taxonomy of behaviour change methods: an intervention mapping approach. Health Psychol Rev.

[19] Verhagen E, Voogt N, Bruinsma A, Finch CF (2014). A knowledge transfer scheme to bridge the gap between science and practice: an integration of existing research frameworks into a tool for practice. Br J Sports Med.

[20] VeiligheidNL (2014). Factsheet Sportblessures 2013.

[21] Hreljac A (2005). Etiology, prevention, and early intervention of overuse injuries in runners: a biomechanical perspective. Phys Med Rehabil Clin N Am.

[22] Fields KB, Sykes JC, Walker KM, Jackson JC (2010). Prevention of running injuries. Curr Sports Med Rep.

[23] Dickson TJ, Terwiel FA (2011). Snowboarding injuries in Australia: investigating risk factors in wrist fractures to enhance injury prevention strategies. Wilderness Environ Med.

[24] Niemuth PE, Johnson RJ, Myers MJ, Thieman TJ (2005). Hip muscle weakness and overuse injuries in recreational runners. Clin J Sport Med.

[25] Behm DG, Blazevich AJ, Kay AD, McHugh M (2016). Acute effects of muscle stretching on physical performance, range of motion, and injury incidence in healthy active individuals: a systematic review. Appl Physiol Nutr Metab.

[26] Hébert-Losier K, Holmberg HC (2013). What are the exercise-based injury prevention recommendations for recreational alpine skiing and snowboarding? A systematic review. Sports Med.

[27] Thomson CJ, Carlson SR (2015). Increased patterns of risky behaviours among helmet wearers in skiing and snowboarding. Accid Anal Prev.

[28] Ehrnthaller C, Gebhard F, Kusche H (2014). Typical injuries in snowboarding. Possible prevention strategies. Unfallchirurg..

[29] Russell K, Christie J, Hagel BE (2010). The effect of helmets on the risk of head and neck injuries among skiers and snowboarders: a meta-analysis. CMAJ..

[30] Haider AH, Saleem T, Bilaniuk JW, Barraco RD, eastern Association for the Surgery of trauma injury ControlViolence prevention committee (2012). An evidence-based review: efficacy of safety helmets in the reduction of head injuries in recreational skiers and snowboarders. J Trauma Acute Care Surg.

[31] Cusimano MD, Kwok J (2010). The effectiveness of helmet wear in skiers and snowboarders: a systematic review. Br J Sports Med.

[32] Hildebrandt C, Mildner E, Hotter B, Kirschner W, Höbenreich C, Raschner C (2011). Accident prevention on ski slopes - perceptions of safety and knowledge of existing rules. Accid Anal Prev..

[33] Fogg BJ. A behaviour model for persuasive design. In proceedings of the 4th international conference on persuasive technology: ACM; 2009. p. 40.

[34] Schaalma H, Kok G, Meertens RM. Intervention mapping. In J. Brug, H. Schaalma, G. Kok, R.M Meertens en, H.T. van der Molen (red.), Gezondheidsvoorlichting en gedragsverandering: een planmatige aanpak (pp.73–88). 2001 Assen: Van Gorcum.

[35] Kools M, van de Wiel MW, Ruiter RA, Cruts A, Kok G (2006). The effect of graphic organisers on subjective and objective comprehension of a health education test. Health Educ Behav.

[36] Smith RM (2008). Conquering the content: a step-by-step guide to web-based course development.

[37] Prochaska JO, Redding CA, Evers KE, Glanz B, Rimer BK, Viswanath K (2008). The transtheoretical model and stages of change. Health behavior and health education.

[38] McAlister AL, Perry CL, Percel GS, Glanz B, Rimer BK, Viswanath K (2008). How individuals, environments, and health behaviours interact. Social cognitive theory. Health behavior and health education.

[39] Bandura A (1997). Self-efficacy: the exercise of control.

[40] Rogers EM (2003). Diffusions of innovations.

[41] Lustria ML, Cortese J, Noar SM, Glueckauf RL (2009). Computer-tailored health interventions delivered over the web: review and analysis of key components. Patient Educ Couns.

[42] Pas HIMFL, Bodde S, Kerkhoffs GMMJ, Pluim B, Tiemessen IJH, Tol JL, Verhagen E, Gouttebarge V (2018). Systematic development of a tennis injury prevention programme. BMJ Open Sport Exerc Med.

[43] Glasgow RE, Vogt TM, Boles SM (1999). Evaluating the public health impact of health promotion interventions: the RE-AIM framework. Am J Public Health.

[44] Kelly MB, Barker M (2016). Why is changing health-related behaviour so difficult?. Public Health.

[45] van Mechelen W, Hlobil H, Kemper HC (1992). Incidence, severity, aetiology and prevention of sports injuries. A review of concepts. Sports Med.

