# Supplementary material for: Stimulating injury-preventive behaviour in sports: the systematic development of two interventions
Source: BMC Sports Sci Med Rehabil. 2019 Oct 21;11:26. doi: 10.1186/s13102-019-0134-8 (PMC6805664; doi:10.1186/s13102-019-0134-8)
Supplement: Supplementary file 1 — Additional file 1. Questionnaire target population Running. [file 13102_2019_134_MOESM1_ESM.pdf]

Questionnaire Running

Demographic characteristics

1. What is your gender?
O man
O woman

2. What is your age?
O Under 20 years of age
O 20-30 years
O 31-40 years
O 41-50 years
O 51-60 years
O Older than 60 years

3. What is the most important reason for you to start running?
O I want to improve my condition or fitness.
O I want to lose weight.
O Running is a relaxation / outlet for me.
O I run because of the social contacts.
O Participation in running event (s).
O Sports in the great outdoors.
O I want to continue to challenge myself, push my limits.
O Running is the basis for my other sports practice.
O Other, namely:

4. Are you a member of a running club or running group?
Oh yes
Oh no

5. How much running experience do you have?
O Less than 6 months.
O 6 to 12 months (0.5 - 1 year).
O 12 to 18 months (1-2 years)
O 18 to 24 months (1.5 - 2 years).
O More than 2 years.

6. How do you estimate your own level?
O Inexperienced runner (beginner)
O A little experienced runner
O Reasonably experienced runner
O Experienced runner (advanced)
O Very experienced runner (expert)

7. Which of the following situations applies to you? (Multiple answers possible)
O I am guided by a trainer while running.
O I train individually.
O I train as a group.
O I train with friend (s) (running buddies).
O I am active in a running clinic such as Start to Run.
O I use a running application for a smartphone.
O I use a sports watch with GPS.
O I use a heart rate monitor.
O I use an activity tracker.

8. Indicate on a scale of 1 to 10 how much you estimate your risk of injury during running. The 1 stands for no risk at all and the 10 for very high risk.

9. Indicate how fit you are on a scale of 1 to 10.
The 1 stands for not at all fit and the 10 for very fit.

10. How many times a week do you exercise in an average week? Sports other than running can also be counted.
O 0-1 times a week (I exercise irregularly)
O once a week
O twice a week
O 3 times a week
O 4 times a week or more

11. Imagine you have an important appointment on the 5th floor of an office and the elevator is out of use. Which situation then applies to you most?
O I do not start up five flights of stairs.
O I walk upstairs at a slow pace, halfway I take a break and then walk on. At the top I need a few minutes to catch my breath.
O I walk up at a slow pace in one go. At the top I need a few minutes to catch my breath.
O I walk upstairs at a leisurely pace. At the top I need a few minutes to catch my breath.
O I walk up at a leisurely pace in one go and do not get out of breath.
O I walk up at a brisk pace. At the top I need a few minutes to catch my breath.
O I walk up at a brisk pace and don't get out of breath.
O I am able to run up the stairs without getting out of breath.
O Other, namely:

General / Design

12. Suppose you receive advice from us that is tailored to your personal situation. What do you expect from this advice (multiple answers possible)
The advice must:
O be brief and to the point, with a reference to additional information.
O be provided with an extensive explanation.
O be directly applicable.
O being provided with practical tools to take action.
O fit in with my personal situation.
O be given during a personal conversation with a specialist in the field of sports injury prevention during running.
O receive a follow-up / evaluation.
O Other, namely:

13. Which device would you like to receive advice from?
O Via my smartphone
O Via my tablet
O Via my laptop / PC
O Via all these devices.

14. In which form do you prefer to receive your advice? (Multiple answers possible.)
O Text
O Drawings
O Photos
O Audio
O Video
O Other, namely:

Content

15. Indicate to what extent you agree with the following statements.
(Strongly agree - Strongly disagree)
• I find injury prevention important when running.

• Everyone can run.
• Running is healthy for everyone.
• Injuries during running cannot be prevented.
• I find it difficult to start running again if I have been injured for a few weeks.

16. How much do you know about injury prevention in running?
O I have lots of knowledge
O I have a lot of knowledge
O I have sufficient knowledge
O I have little knowledge
O I have very little knowledge

17. Which measures to prevent running injuries do you apply?
……………………………………………… ..

18. What do you think is the most important measure to prevent running injuries?
………………………………………………………………………………………………

……………………………………………………………………………………………….

19. What are the reason (s) for you to pay attention to injury prevention?
O An earlier injury while running.
O Need to protect myself against injuries (prevention of injuries).
O Others who advise me to do injury prevention.
O Others who also do injury prevention.
O Other, namely

20. Do you use a running schedule while running?
O Yes
O No (go to question 21)

21. Where some runners do exactly what the schedule / coaching prescribes in an app, others do not always follow it up. To what extent do you adhere to the schedule / coaching instructions?
O Always
O Usually
O Sometimes yes / sometimes no
O Usually not
O Never

22. How do you deal with pain before, during or after running? Answer the following statements. (Agree Disagree)
O I have never had pain before, during or after running.
O Morning pain is no reason to adjust the next workout.
O I do not train further with pain complaints at the start of a training.
O For short-term (or minor) pain complaints after a workout, I take extra rest.
O I find it difficult to properly assess pain complaints.
O An injury only occurs if the pain symptoms persist.

23. The reason for running is different for many people. The motivation strength for running also varies from person to person. To what extent are you motivated to run?
O Very motivated
O Motivated
O Reasonably motivated
O A little motivated
O Not motivated

24. In which situations do you recognize yourself? (Multiple answers possible.)
O If my running buddy cancels, I often stay at home.
O Despite good intentions, running often fails me.
O If I have planned to go running, then I will also go running.

O I want to keep improving my personal performance.
O I do running because it has to be from the doctor.
O For running I put everything aside.
O Even if I don't feel fit, I go running.

25. If you receive advice on sports injury prevention that is tailored to your personal situation, what should that advice focus on? (multiple answers possible)
I want to:
O know why I have to do injury prevention while running.
O know how to properly assess risks.
O know with which measures I can prevent injuries while running.
O know how to build up my training.
O want to receive specific tips about injury prevention during running.
O Other, namely:

26. You have indicated what the advice should focus on. Which of these topics do you find most important?
O Reasons why injury prevention is important.
O Risks while running.
O Information about how injuries can be prevented.
O Concrete tips on injury prevention during running.
O Concrete tips and exercises for a good training structure.
O Other, namely:

27. Finally, to what extent do you expect that the advice that is tailored to your personal situation can contribute to actual changes in your behavior?

I expect such advice:
O can contribute a lot.
O can contribute a lot.
O little can contribute.
O very little can contribute.
O cannot contribute.
