# Supplementary material for: Stimulating injury-preventive behaviour in sports: the systematic development of two interventions
Source: BMC Sports Sci Med Rehabil. 2019 Oct 21;11:26. doi: 10.1186/s13102-019-0134-8 (PMC6805664; doi:10.1186/s13102-019-0134-8)
Supplement: Supplementary file 2 — Additional file 2. Questionnaire target population Skiing. [file 13102_2019_134_MOESM2_ESM.pdf]

Demographic characteristics

1. What is your gender?
O man
O woman

2. What is your age?
O 17 years or younger
O 18-24 years
O 25-29 years
O 30-34 years
O 35-39 years
O 40-44 years
O 45-49 years
O 50-54 years
O 55 years and older

3. Which winter sports do you practice?
O skiing
O snowboarding
O Skiing and snowboarding
O None of these ◊ excluded from participation in the study

4. How much winter sports experience do you have?
O 0-2 weeks
O 3-5 weeks
O 6-9 weeks
O 10 weeks or more
O I am a ski instructor or snowboard instructor
◊ How many weeks do you work as an instructor per year? ......

5. How many weeks have you had ski and / or snowboard lessons?
O 0-2 weeks
O 3-5 weeks
O 6-9 weeks
O 10 weeks or more

6. On average how many weeks a year are you active in winter sports?
O less than 1 week a year
O 1 week a year
O 2 weeks a year
O 3 or more weeks per year

7. How do you estimate your own level?
O Inexperienced (beginner)
O Experience a bit
O Reasonably experienced
O Experienced (advanced)
O Very experienced (expert)

8. What does your preparation for your winter sports holiday consist of? (Multiple answers possible.)
O I check the snow depths in my ski area.
O I follow the SneeuwFit program of the Dutch Ski Association.
O I take a number of lessons at a ski slope or club in the Netherlands.
O I have my winter sports equipment checked for defects and corrected if necessary.
O I do muscle strengthening exercises.
O I ensure that I am physically fit.
O I arrange the rental of winter sports equipment.
O I look in advance at the slopes where I am going.

O I pack my bag, but do not prepare further.
O I do enough sport already.
O Other, namely:

9. How high do you rate your risk of injury during winter sports on a scale of 1 to 10, with the 1 representing no risk at all and the 10 representing a very high risk?

10. How do you estimate your own fitness? What rating would you give yourself on a scale of 1 to 10, where the 1 stands for not at all fit and the 10 for very fit?

11. How many times a week do you practice sports? Assume an average week in the year.
O 0-1 times a week
O once a week
O twice a week
O 3 times a week
O 4 times a week or more
O I don't do any sport

12. Suppose you have an important appointment on the 5th floor of an office and the elevator is out of use. Which situation applies to you most?
O I do not start up five flights of stairs.
O I walk upstairs at a slow pace, halfway I take a break and then walk on. At the top I need a few minutes to catch my breath.
O I walk up at a slow pace in one go. At the top I need a few minutes to catch my breath.
O I walk upstairs at a leisurely pace. At the top I need a few minutes to catch my breath.
O I walk up at a brisk pace. At the top I need a few minutes to catch my breath.
O I walk up at a brisk pace and don't get out of breath.
O I am able to run up the stairs without getting out of breath.
O Other, namely:

Sports injury prevention advice tool

13. Suppose you receive advice from us about sports injury prevention during winter sports that is tailored to your personal situation. What do you expect from this advice? (Multiple answers possible.) The advice must:
O be brief and to the point, with a reference to additional information.
O be provided with an extensive explanation.
O be directly applicable.
O being provided with practical tools to take action.
O be given during a personal conversation with a specialist in the field of sports injury prevention when skiing or snowboarding.
O otherwise, namely:

14. Which device would you like to receive advice from?
O Via my smartphone
O Via my tablet
O Via my laptop / PC
O All these devices
O Other, namely:

15. In which form do you prefer to receive your advice? (Multiple answers possible.)
O Text
O Drawings
O Photos
O Audio
O Video
O Other, namely:

16. What do you think is the most suitable moment for advice on sports injury prevention in winter sports that is tailored to your personal situation?
O If I am going to book my winter sports.
O When I prepare for my winter sport.
O At the start of the winter sports season.
O At the start of my winter sports holiday.
O Other, namely:

Content advisory tool sports injury prevention

17. Answer the following statements: (Strongly agree - completely disagree.)
• I consider sports injury prevention important for skiing and snowboarding.
• I pay attention to sports injury prevention before going on a winter sport.
• Injuries during winter sports cannot be prevented.

18. We want to give winter sports enthusiasts advice on injury prevention in skiing and snowboarding that is tailored to their personal situation. How do you estimate your knowledge about injury prevention measures during / at profit sports?
O Excellent
O Good
O Sufficient
O reasonable
O Moderate

19. Which injury prevention measures do you apply yourself while skiing or snowboarding? (Multiple answers possible.)
O I am wearing a winter sports helmet.
O I use wrist protectors.
O I use a back protector.
O I prepare myself physically for winter sports.
O I wear ski goggles or goggles.
O I use sunscreen.
O I have my equipment checked before the winter sports season and adjusted if necessary.
O I take one or more lessons.
O Other, namely ……………………….

(Ask if physical preparation is not ticked in question 19)
20. You apply one or more injury prevention measures, but do not prepare yourself physically for the winter sports holiday. Can you indicate why not?
O I do enough sports / I find myself fit enough.
O It's a vacation.
O I would not know what to do.
O I wouldn't know how to do that.
O Other, namely:

21. What is a reason for you to do injury prevention during / when skiing or snowboarding or what could be a reason for you?
O An earlier injury during skiing / snowboarding.
O Need to protect myself against injuries.
O If it is recommended to me by others.
O An earlier accident or an earlier collision on the slopes.
O If others also do injury prevention.
O I want to set a good example for my children.
O Easy to apply measures.
O Other, namely

22. If you receive advice on sports injury prevention that is tailored to your personal situation, what should that advice focus on? (Multiple answers possible.)

I want to:

O know why I have to do injury prevention while skiing or snowboarding.
O know how to properly assess risks.
O know with which measures I can prevent injuries during skiing or snowboarding.
O know how to prepare myself physically for a winter sports holiday.
O receive concrete tips about injury prevention during skiing or snowboarding.
O Otherwise, namely:

23. You have indicated what the customized advice should focus on. Which of these topics do you find most important?
O Reasons why injury prevention is important.
O Risks while skiing or snowboarding.
O Information about how injuries can be prevented.
O Concrete tips on injury prevention during skiing or snowboarding.
O Concrete tips and exercises for good physical preparation for winter sports.
O Otherwise, namely:

24. Finally, to what extent do you expect that the advice that is tailored to your personal situation can contribute to actual changes in your behavior?

I expect such advice:
O can contribute a lot.
O can contribute a lot.
O little can contribute.
O very little can contribute.
O cannot contribute.
