# Supplementary material for: Stimulating injury-preventive behaviour in sports: the systematic development of two interventions
Source: BMC Sports Sci Med Rehabil. 2019 Oct 21;11:26. doi: 10.1186/s13102-019-0134-8 (PMC6805664; doi:10.1186/s13102-019-0134-8)
Supplement: Supplementary file 3 — Additional file 3. Roadmap Focus group sessions Running. [file 13102_2019_134_MOESM3_ESM.pdf]

Roadmap Tailor-made Advice - Focus group Running

Part A Determinants and expert recommendations (6 p.m. - 7 p.m.)
1. Short welcome, also on behalf of Athletics Union (5min)
2. Just fill in (again) selection questions on form (5min)
3. Proposal round (20 min)
a. Name, why do you run? Best running experience? Why do you participate in this focus group?
b. Answer questions (form)
c. Where would you place yourself in the quadrant? / explanation
d. Discuss audit questions.
4. Group discussion (15min)
a. To whom is this recognizable? Meaningful layout?
b. Does this short self-analysis motivate you to get started (injury prevention)?
c. Why yes / no? How can we support your motivation (what could work / open questions?)
d. How to contribute to increasing the intention to change? (what could work / open questions?)
5. Advice expert based on quadrant (display on screen) (15min)
a. What can you do with that? / appealing, why not?
b. How could you translate these recommendations into action behavior (what would cause you to get started?)
♣ Spontaneously (what could work for you to really do something?)
♣ Helped; brief explanation of existing mechanisms + example:
• Tailor-made (tailoring)> such as dividing yourself into a quadrant and then receiving advice
• Reduction (baby steps to new behavior)> extensive advice versus very simple / short / applicable. What would you do with it? What
• Suggestions on how to take steps (making advice very practical)
• Reminders / compliment (follow 1st steps and receive a virtual compliment, or from others)

Short break (19.05-19.15)

Part B Form and examples (19.15 - 19.50)
6. Form
o text / video / app (preference? round)
o timing: when received / when admissible?
o Through which channels / other specs
o Show some examples of existing applications (benchmark / responses)

7. Conclusion
o Comments / questions
o Next steps
o Participation cocreatiegroep?

URLs example apps

[internal information]

Target session:
Gaining deeper insight into:
1. Determinants (self-assessment) & how to contribute to intention to change?
2. How to promote action behavior?
3. Which form / channels / specifications fit best?

Intended result
• Overview of user requirements for 3/4 steps:
o self-insight / determination
o advice
o action
o (feedback / follow)
• "What would you like for each phase?"
