# Supplementary material for: Stimulating injury-preventive behaviour in sports: the systematic development of two interventions
Source: BMC Sports Sci Med Rehabil. 2019 Oct 21;11:26. doi: 10.1186/s13102-019-0134-8 (PMC6805664; doi:10.1186/s13102-019-0134-8)
Supplement: Supplementary file 4 — Additional file 4. Roadmap Focus group sessions Skiing. [file 13102_2019_134_MOESM4_ESM.pdf]

Tailor-made Advice - Focus Group Wintersport

Part A Determinants and expert recommendations (6 p.m. - 7 p.m.)
1. Short welcome, also on behalf of NSv (5min)

2. Just fill in (again) selection questions on form (5min)

3. Proposal round (20 min)
a. name, ski / snowboarder, favorite winter sports location
b. answer questions (form)
c. Where would you place yourself in the quadrant? / explanation

4. Group discussion (15min)
a. To whom is this recognizable? Meaningful layout? (Possibly testing other formats / typologies) / show archetypes
b. Does this short self-analysis motivate you to get started (injury prevention)?
c. Why yes / no? How can we support your motivation (what could work / open questions?)
d. How to contribute to increasing the intention to change? (what could work / open questions?)

5. Do Ski APK Achmea / Plenary.
a. Does this change your self-diagnosis?
b. Are you able to estimate yourself (also) without doing these exercises yourself? Is it motivating?

6. Advice expert based on quadrant (showing on screen) (15min)
a. What can you do with that? / appealing, why not?
b. How could you translate these recommendations into action behavior (what would cause you to get started?)
♣ Spontaneously (what could work for you to really do something?)
♣ Helped; brief explanation of existing mechanisms + example:
• Tailor-made (tailoring)> such as dividing yourself into a quadrant and then receiving advice
• Reduction (baby steps to new behavior)> extensive advice versus very simple / short / applicable. What would you do with it? What
• Suggestions on how to take steps (making advice very practical)
• Reminders / compliment (follow 1st steps and receive a virtual compliment, or from others)

Short break (19.05-19.15)

Part B Form and examples (19.15 - 19.50)
7. Form
o text / video / app (preference? round)
o timing: when received / when admissible?
o Through which channels / other specs
o Show some examples of existing applications (benchmark / responses)
http://50 questions.zilverenkruis.nl/ski-apk/
https://www.wintersport.nl/sneeuwfit/sneeuwfit-online/
[internal information]

Target session:
Gaining deeper insight into:
1. Determinants (self-assessment) & how to contribute to intention to change?
2. How to promote action behavior?
3. Which form / channels / specifications fit best?

Intended result
• Overview of user requirements for 3/4 steps
o self-insight / determination
o advice
o action
o (feedback / follow)
• "What would you like for each phase
